# Editorial: Organoids and 3D culture: bridging tissue complexity in biomedical research

**DOI:** 10.3389/fmed.2026.1959510

**Published:** 2026-08-26

**Authors:** Bianca Vezzani, Ilaria Genovese

**Affiliations:** 1 Department of Integrative Biology and Physiology, University of Minnesota, Minneapolis, MN, United States; 2 Department of Medicine and Surgery, University of Parma, Parma, Italy; 3 Departmental Faculty of Medicine UniCamillus-Saint Camillus International University of Health Sciences, Rome, Italy

**Keywords:** 3D culture, *in vitro*, organoid, spheroids, tissue modeling

One of the major challenges in biomedical research is translating discoveries into clinical approaches. To address this, every discovery must be validated in several models, each with increasing complexity, with the final aim of reproducing human physiology. In this context, accurately modeling human tissue complexity remains a major challenge. Scientists have applied the reductionist approach, breaking the complexity of organisms into their smallest living parts: the cells. Since the beginning of the 20th century, the ability to isolate living cells from organisms has been one of the most important discoveries for translational approaches, enabling the development of two-dimensional (2D) cell culture systems. On the one hand, methodological reductionism has provided fundamental insights into cellular mechanisms and disease biology, but on the other hand, it has always required support from *in vivo* studies on animal models to ensure the translatability of discoveries to human physiology. In fact, 2D cultures have a limited capacity to reproduce tissue architecture because they lack cellular interactions and extracellular matrix organization, and therefore dynamic microenvironmental signals. This limitation stems from the basic principle of cell cultures, which are mainly characterized by a single cell type forced to grow on plastic surfaces, sometimes with the support of extracellular matrix components (such as vitronectin, collagen, poly-lysine) or feeder layers (as for induced pluripotent stem cells, iPSCs). To overcome these limitations, scientists developed protocols that allowed the culture of entire organs, namely organotypic cultures. Compared to traditional 2D cultures, they offer a more physiologically relevant model by preserving tissue–tissue interactions and microenvironments. From the first primordial culture of rabbit organs in the the end of 19th century, organotypic cultures have evolved, maintaining their fundamental principle: being derived from *ex vivo* organs kept in culture, either in small slices or as entire organs, therefore requiring animal models or human subject biopsies (1).

With advances in stem cell biology and growing knowledge of the specific pathways activated during tissue differentiation, researchers have aimed to generate sophisticated three-dimensional (3D) models from cell culture, thereby reducing reliance on animal models as a source for organotypic cultures. Notably, 3D culture systems derived from iPSCs, embryonic stem cells, mesenchymal stem cells, and cancer stem cells have enabled the generation of a large subset of tissue-like and organoid structures. These include the brain, gut/intestine, kidney, cardiac, and sensory organs. Over the last decade, organoids and advanced 3D culture technologies have transformed disease modeling, advanced basic biological research, and enhanced drug discovery platforms, enabling the generation of human cell-based models that more closely recapitulate key aspects of tissue structure and function (2, 3). A major advantage of organoids is their ability to preserve clinically relevant features of human cells, such as genetic mutations, while maintaining complex structure and cellular interactions. In particular, patient-derived organoids have introduced new opportunities to investigate disease biology in a personalized context and to evaluate therapeutic responses using patient-specific models. In oncology, tumor organoids have demonstrated considerable potential to capture aspects of tumor heterogeneity and drug sensitivity, providing complementary approaches to traditional preclinical models and supporting the development of individualized treatment strategies (4, 5). Nevertheless, this rapidly evolving field remains one of the most challenging in cell biology, given the complexity of recreating a bona fide tissue's spatial organization with diverse cell types.

The Research Topic “*Organoids and 3D culture: bridging tissue complexity in biomedical research*” aims to compile advanced protocols for producing 3D cellular cultures and developing them into functional organoids. It emphasizes the importance of organoids as essential and innovative tools for establishing reliable *in vitro* models in biomedicine. The collected contributions show that 3D models are increasingly evolving beyond their initial use as experimental platforms for studying development and disease, becoming valuable tools for therapeutic discovery, functional testing, and personalized medicine.

In this Research Topic, Acar presents a literature review on the growing role of patient-derived tumor organoids in precision oncology, emphasizing their potential as “personalized therapeutic avatars” that are fundamental to understanding tumor behavior and predicting therapeutic responses. van den Berg et al. support Acar's arguments, providing evidence for the reliable use of organoids to model pheochromocytomas, a rare neuroendocrine tumor. This approach marks an important step toward integrating patient-specific biological information into treatment decision-making. In parallel with organoids, organotypic culture techniques continue to advance, as shown in the article by Fuchs et al., where the authors develop a whole-brain slice model for glioblastoma investigation, representing an important technological advance toward preserving tissue architecture and tumor–microenvironment interactions. Lastly, Galluzzi et al. review the contribution of iPSC-derived organoids as a promising human-based platform for amyotrophic lateral sclerosis (ALS) research, highlighting how patient-derived 3D systems can provide novel insights into disease mechanisms and therapeutic opportunities.

Collectively, the present article collection highlights how organoids derived from human cells serve as valuable experimental resources, allowing the precise reproducibility of tumor peculiarity and granting physiological modeling of neurological disorders, where access to human tissue and disease-relevant models remains a major challenge. However, despite the remarkable progress described, organoid techniques still face several challenges to achieve broader clinical translation. These include variability between organoid preparations, incomplete maturation, limited integration of vascular and immune components, and the absence of universally accepted standards for production and characterization. Addressing these limitations will require multidisciplinary efforts combining stem cell biology, bioengineering, biomaterials, computational approaches, and multi-omics technologies (6). The future development of organoid platforms will likely depend on their integration into increasingly sophisticated translational pipelines. Advances in engineering approaches, high-content imaging, molecular profiling, and computational modeling are expected to further improve their predictive capacity and facilitate their incorporation into drug development workflows and clinical research. The ultimate impact of these technologies will not only depend on their ability to reproduce tissue complexity, but also on their capacity to generate clinically actionable information.
